# Discrimination and prediction of the origin of Chinese and Korean soybeans using Fourier transform infrared spectrometry (FT-IR) with multivariate statistical analysis

**DOI:** 10.1371/journal.pone.0196315

**Published:** 2018-04-24

**Authors:** Byeong-Ju Lee, Yaoyao Zhou, Jae Soung Lee, Byeung Kon Shin, Jeong-Ah Seo, Doyup Lee, Young-Suk Kim, Hyung-Kyoon Choi

**Affiliations:** 1 College of Pharmacy, Chung-Ang University, Seoul, Republic of Korea; 2 National Agricultural Products Quality Management Service, Gimcheon, Republic of Korea; 3 School of Systems Biomedical Science, Soongsil University, Seoul, Republic of Korea; 4 Department of Bio and Fermentation Convergence Technology, Kookmin University, Seoul, Republic of Korea; 5 Department of Food Science and Engineering, Ewha Womans University, Seoul, Republic of Korea; College of Agricultural Sciences, UNITED STATES

## Abstract

The ability to determine the origin of soybeans is an important issue following the inclusion of this information in the labeling of agricultural food products becoming mandatory in South Korea in 2017. This study was carried out to construct a prediction model for discriminating Chinese and Korean soybeans using Fourier-transform infrared (FT-IR) spectroscopy and multivariate statistical analysis. The optimal prediction models for discriminating soybean samples were obtained by selecting appropriate scaling methods, normalization methods, variable influence on projection (VIP) cutoff values, and wave-number regions. The factors for constructing the optimal partial-least-squares regression (PLSR) prediction model were using second derivatives, vector normalization, unit variance scaling, and the 4000–400 cm^–1^ region (excluding water vapor and carbon dioxide). The PLSR model for discriminating Chinese and Korean soybean samples had the best predictability when a VIP cutoff value was not applied. When Chinese soybean samples were identified, a PLSR model that has the lowest root-mean-square error of the prediction value was obtained using a VIP cutoff value of 1.5. The optimal PLSR prediction model for discriminating Korean soybean samples was also obtained using a VIP cutoff value of 1.5. This is the first study that has combined FT-IR spectroscopy with normalization methods, VIP cutoff values, and selected wave-number regions for discriminating Chinese and Korean soybeans.

## Introduction

The soybean (*Glycine max*) is a useful plant crop with high lipid and protein contents [1]. Soybeans can be used to produce soybean oil, as a protein source, or as a good source of nutrients. They are also pharmacologically active, with these effects originating from their constituent isoflavones [2]. The beneficial health effects of soybean isoflavones include reducing the risks of cardiovascular problems [3,4], cancer [5–7], and osteoporosis [8,9].

In Korea, soybeans are cooked and used to prepare foodstuffs such as doenjang (fermented soybean paste), cheonggukjang (fast-fermented soybean paste), and gochujang (fermented red pepper paste) [10]. Soybeans are frequently used in Korean cuisine. However, there are many cases where the country of origin of the beans is unclear, and relatively inexpensive foreign soybeans are often imported and labeled as Korean soybeans. The National Agricultural Products Quality Management Service introduced an agricultural food country-of-origin labeling system in 1991 to protect domestic agricultural producers and consumers [11]. Soybeans have been included in that system since 2017, and merchants must now indicate the origin of any soybeans that they advertise for sale [12]. This situation means that technology for discriminating Chinese and Korean soybean is needed.

The quality of soybeans depends on several factors such as their variety and where they were cultivated, and these factors must be considered when determining where particular soybeans originate from. However, it is difficult to consider all soybean varieties because there are hundreds of varieties spread over a vast area [13]. We assumed that soybeans cultivated for thousands of years within a particular region would have become well adapted to the local environmental conditions, and hence that the soybeans could be discriminated based on geographical factors rather than varietal differences.

Metabolomics can be used to discriminate genetic and environmental differences based on the comprehensive profiling and analysis of plant metabolites [14]. This can be implemented using established tools such as gas chromatography/mass spectrometry, nuclear magnetic resonance (NMR) spectroscopy, liquid chromatography/mass spectrometry, Fourier-transform infrared (FT-IR) spectroscopy, and direct-infusion mass spectrometry [15]. These tools can be used to discriminate the geographical origin of plants. For example, a method employing a so-called electronic nose and combined gas chromatography/mass spectrometry/olfactometry with principal-components analysis has been used to discriminate the geographical origin of chrysanthemum flower teas [16]. ^1^H-NMR spectroscopy has been combined with statistical analysis to discriminate the geographical origin of Chinese, Indian, and Korean sesame oils [17]. Four different geographical origins of *Lycium barbarum* fruit (China, Mongolia, and two locations in Tibet) were discriminated using liquid chromatography coupled with quadrupole time-of-flight mass spectrometry for metabolite profiling [18]. Near-infrared reflectance (NIR) spectroscopy has been used to discriminate Korean soybeans from soybeans of various origins [19].

We chose FT-IR spectroscopy for the present study because it is a fast, convenient, and nondestructive analytical tool. These characteristics make FT-IR spectroscopy suitable for the rapid identification of foods and agricultural products [20]. However, the physical characteristics of samples (particle size and thickness) affect the obtained FT-IR spectra [21,22], and so the obtained raw data need to be normalized. Four normalization methods can be applied to FT-IR spectral data: area normalization, amide normalization, minimum-maximum (min-max) normalization, and vector normalization. Constructing more-precise prediction models for the discrimination of Chinese and Korean soybeans requires suitable normalization and scaling methods to be determined, and then a prediction model selected by comparing the predictive power of each cutoff for the variable influence on projection (VIP).

NMR spectroscopy has previously been used to discriminate between soybeans originating from China and Korea [23], while NIR spectroscopy was used to discriminate Korean soybeans and soybeans of various origins [19]. However, the present study is the first to investigate a prediction model that can discriminate between Chinese and Korean soybeans using FT-IR spectroscopy combined with scaling methods, optimal normalization methods, the selection of an appropriate wave-number region, and a VIP cutoff value.

## Materials and methods

### Soybean materials and sample preparation

**Fig 1:**
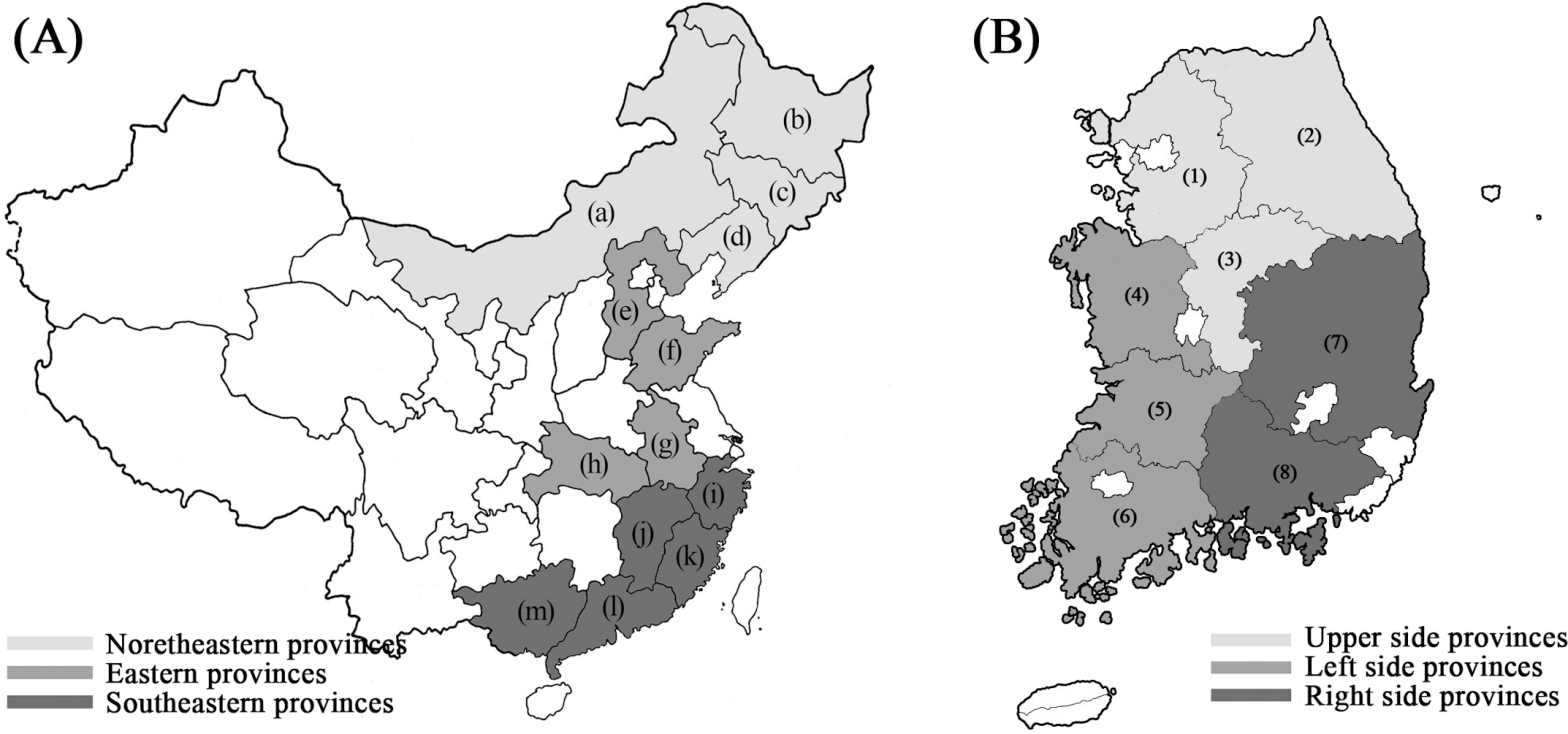
Map showing the origin of the Chinese and Korean soybeans used in the experiments. (A) Map of China. The Chinese provinces were divided into three regions: northeastern, eastern, and southeastern. The northeastern region comprises four provinces: (a) Neimenggu, (b) Heilongjiang, (c) Jilin, and (d) Liaoning. The eastern region comprises four provinces: (e) Hebei, (f) Shandong, (g) Anhui, and (h) Hubei. The southeastern region comprises five provinces: (i) Zhejiang, (j) Jiangxi, (k) Fujian, (l) Guangdong, and (m) Guangxi. (B) Map of South Korea. The South Korean provinces were divided into three regions: upper, left side, and right side. The upper region comprises three provinces: (1) Gyeonggi-do, (2) Gangwon-do, and (3) Chungcheongbuk-do. The left-side region comprises three provinces: (4) Chungcheongnam-do, (5) Jeollabuk-do, (6) and Jeollanam-do. The right-side region comprises two provinces: (7) Gyeongsangbuk-do and (8) Gyeongsangnam-do.

**Fig 2:**
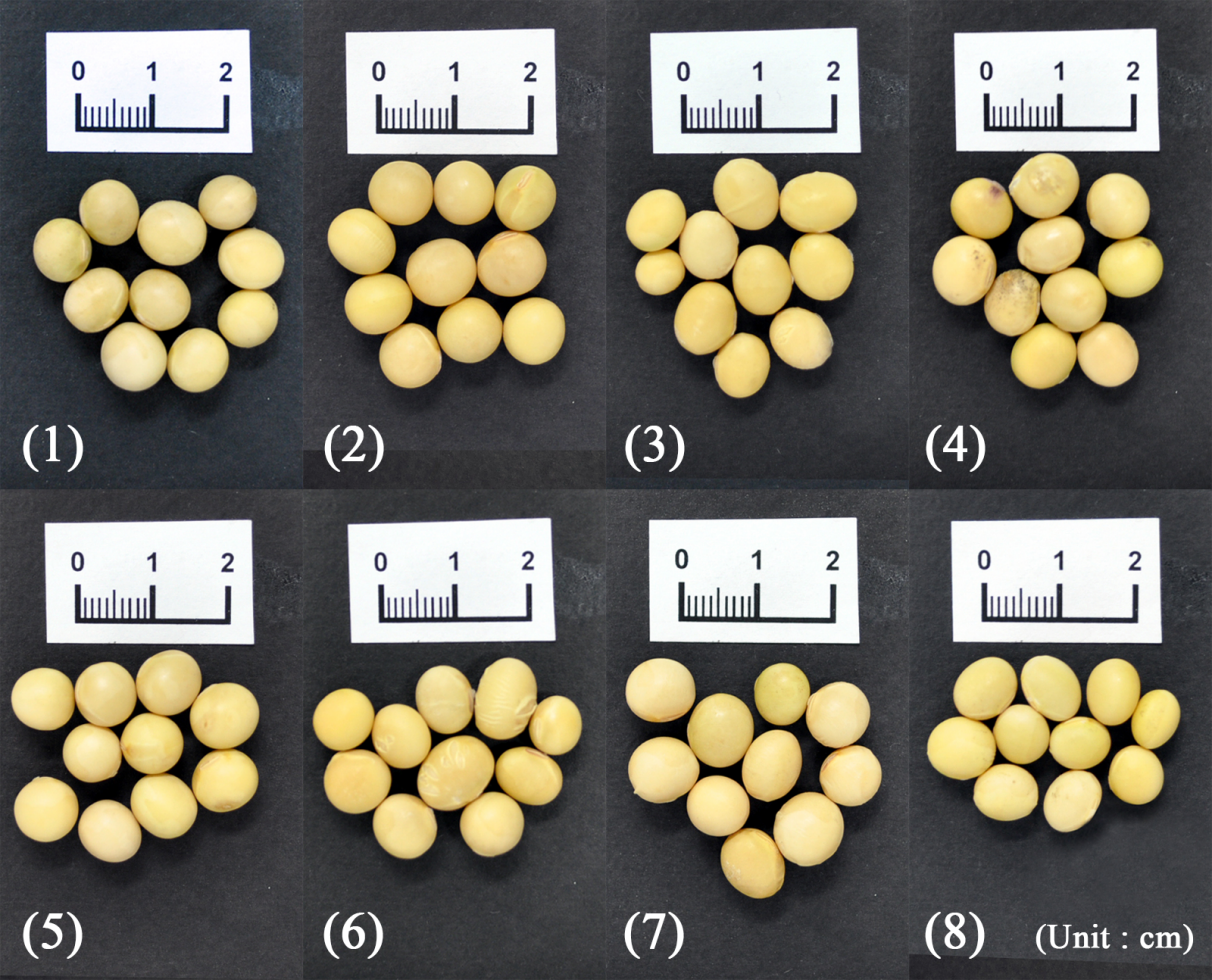
Morphological characteristics of the eight Korean soybean samples. (1) Gyeonggi-do Anseong, (2) Gangwon-do Yeongwol, (3) Chungcheongbuk-do Eumseong, (4) Chungcheongnam-do Cheonan, (5) Jeollabuk-do Imsil, (6) Jeollanam-do Yeonggwang, (7) Gyeongsangbuk-do Uiseong, and (8) Gyeongsangnam-do Geochang.

**Fig 3:**
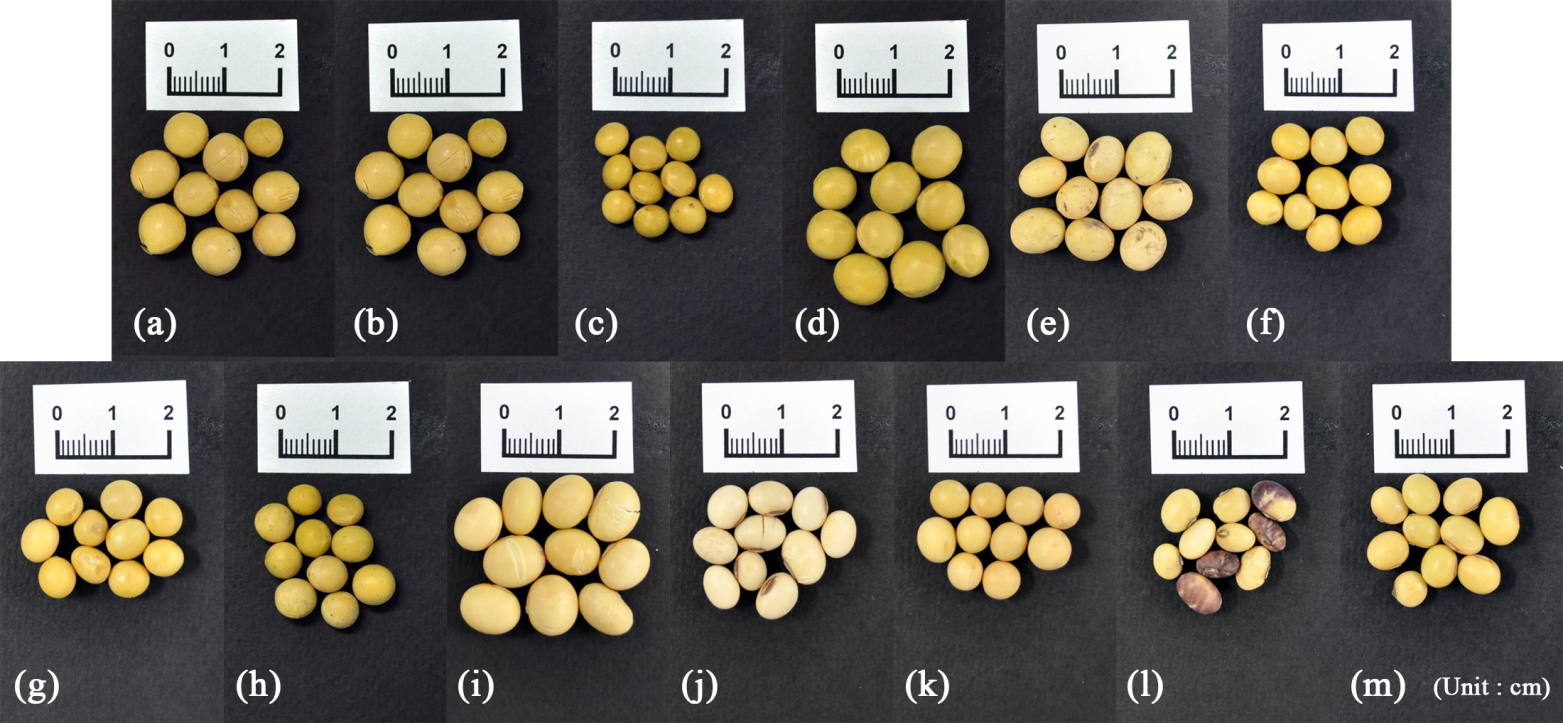
Morphological characteristics of the 13 Chinese soybean samples.

As shown in Fig 1, 21 soybean samples collected from Korea (8 samples) and China (13 samples) were prepared for analysis by FT-IR spectroscopy. Korean soybeans were obtained from the National Agricultural Products Quality Management Service (Fig 2, S1 Table), and Chinese soybeans were obtained from a Chinese market (Fig 3, S1 Table). The 8 Korean soybean samples had been cultivated in Gyeonggi-do Anseong, Gangwon-do Yeongwol, Chungcheongbuk-do Eumseong, Chungcheongnam-do Cheonan, Jeollabuk-do Imsil, Jeollanam-do Yeonggwang, Gyeongsangbuk-do Uiseong, and Gyeongsangnam-do Geochang. The 13 Chinese soybean samples were obtained from Neimenggu, Heilongjiang, Jilin, Liaoning, Hebei, Shandong, Anhui, Hubei, Zhejiang, Jiangxi, Fujian, Guangdong, and Guangxi. The provinces, cities, and geographic coordinates of soybean samples were listed in S1 Table. Ten individual soybeans were randomly selected for each region, frozen rapidly in liquid nitrogen, ground into a fine powder using a mixer, and stored at −80°C before further analysis.

### FT-IR spectroscopy analysis and spectral data preprocessing

Soybean powder was loaded onto an FT-IR spectrometer (NICOLET iS50, Thermo Fisher Scientific, Kyoto, Japan) equipped with an attenuated total reflection (ATR) accessory for recording the FT-IR spectra. The OMNIC program (version 8.2.0.387, Thermo Scientific, Waltham, Massachusetts, USA) was used to obtain all of the FT-IR spectra. Sixty-four scans were recorded in order to obtain average analytical results and enhance the signal-to-noise ratio. Each spectrum was scanned between 4000 and 400 cm^–1^ and had a spectral resolution of 4 cm^–1^.

The following four normalization methods that are widely used in FT-IR spectroscopy analysis were used to process the FT-IR spectra: area normalization, min-max normalization, amide normalization, and vector normalization. In vector normalization, all spectra are converted from transmittance to absorbance, and the FT-IR absorbance spectra were converted into first and second derivatives using the Savitzky-Golay derivative with nine smoothing points in OMNIC. For vector normalization, the absorbance values of FT-IR spectral data were divided by the Euclidean norm to calculate the vector normalization value. For the other normalization processes, all spectra were converted from transmittance to absorbance, and then ATR correction was applied using OMNIC. For area normalization, each absorbance value at a specific wave number was divided by the total (integrated) absorbance area of the spectrum. For min-max normalization, each absorbance value was divided by the difference between the highest and lowest absorbance values. For amide normalization, each absorbance value was divided by the difference between the highest amide band and the lowest absorbance value.

### Multivariate statistical analysis

After the FT-IR spectral data had been normalized, we used the SIMCA-P+ software (version 13.0, Umetrics, Umeå, Sweden) to carry out multivariate statistical analysis. Partial-least-squares discriminant analysis (PLS-DA), partial-least-squares regression (PLSR), and hierarchical cluster analysis (HCA) were conducted using SIMCA-P+. Both the single linkage method and Ward’s clustering method were employed to carry out HCA. Cross-validation and permutation tests were applied to the PLS-DA and PLSR models. Cross-validation was performed to evaluate the predictability of the models and to prevent overfitting. The models were evaluated using the R^2^Y and Q^2^Y parameters as obtained by cross-validation. Permutation tests were conducted 20 times using SIMCA-P+. Permutation test parameters such as the R^2^Y and Q^2^Y intercepts were obtained to evaluate the statistical significance of the models.

## Results and discussion

### Band assignment of the FT-IR spectra

**Fig 4:**
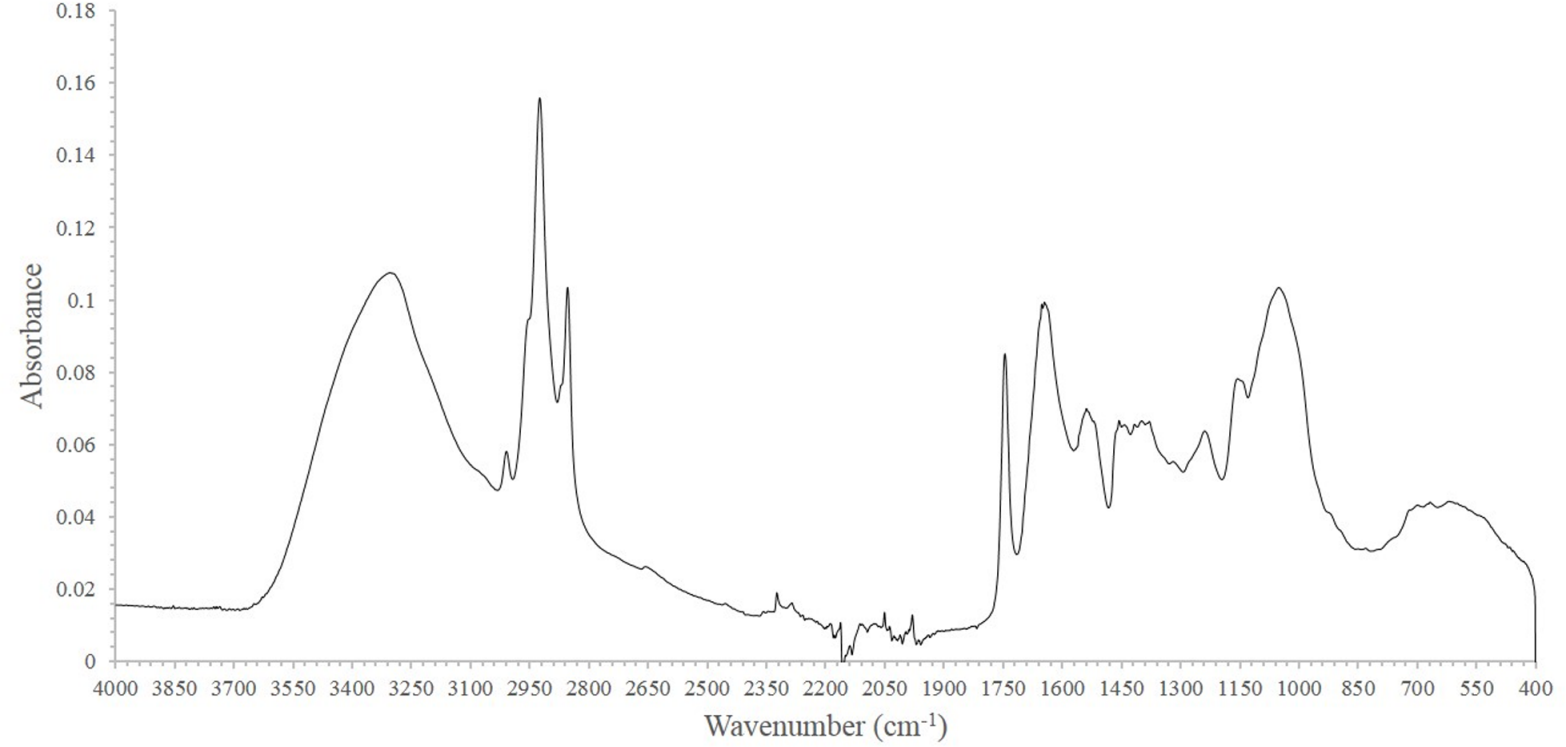
Representative FT-IR spectral data of soybeans from Neimenggu province.

FT-IR spectral data were obtained for each soybean sample. A representative FT-IR spectrum—from the sample from Inner Mongolia Autonomous Region province in China—is shown in Fig 4, which contained 12 noticeable bands that could be assigned as follows (Table 1):

1. One at 3304 cm^–1^ due to N-H protein stretching [24].
2. One at 3009 cm^–1^ due to C = H stretching of unsaturated lipids [25].
3. One at 2925 cm^–1^ due to asymmetric C-H stretching of lipids [26].
4. One at 2854 cm^–1^ due to symmetric C-H stretching of lipids [26].
5. One at 1745 cm^–1^ due to C = O stretching of lipids [27].
6. One at 1645 cm^–1^ due to C-O and C-N protein stretching [24]. This is known as the amide I band and is the main amide band.
7. One at 1538 cm^–1^ due to C-N stretching and N-H bending modes of protein. This is known as the amide II band [24].
8. One at 1456 cm^–1^ due to CH_2_ bending of lipids [26].
9. One at 1398 cm^–1^ due to CH_3_ bending of protein and COO^−^symmetric stretching of fatty acids and amino acids [25,28].
10. One at 1239 cm^–1^, which is the amide III band that contains contributions from PO^2–^ asymmetric stretching [28].
11. One at 1155 cm^–1^ due to CO-O-C asymmetric stretching of cholesterol ester and C-O stretching of oligosaccharides and triacylglycerols [25,29].
12. One at 1051 cm^–1^ due to C-O stretching of starch [30].

**Table 1:** FT-IR spectrum band assignments of soybeans cultivated in Neimenggu province.

Wavenumber (cm^-1^)	Vibration	Suggested biomolecular assignment	Reference
4000–3500	O-H stretching	H_2_O	[31]
3304	N-H stretching	Amide A (protein)	[24]
	N-H and O-H stretching	Polysaccharides, proteins	[26]
3009	C = H stretching	Unsaturated lipids	[25]
2925	C-H stretching (asym)	Lipids (mainly), proteins, carbohydrates	[26]
2854	C-H stretching (sym)	Lipids (mainly), proteins, carbohydrates	[26]
2442–2208	O-C-O stretching	CO_2_	[31]
1745	C = O stretching	Lipids	[27]
1645	C-O, C-N stretching	Amide I (protein)	[24]
1538	C-N stretching, N-H bending	Amide II (protein)	[24]
1456	CH_2_ bending	Lipids	[26]
1398	CH_3_ bending	Proteins	[28]
	COO^-^ stretching (sym)	Fatty acids, amino acids	[25]
1239	PO^2-^ stretching (asym)	Amide III	[28]
1155	CO-O-C stretching (asym)	Cholesterol ester	[25]
	C-O stretching	Oligosaccharides, triacylglycerols	[29]
1051	PO^2-^ stretching (sym)	Nucleic acids	[28]
	C-O stretching	Starch	[30]
914–600	O-C-O bending	CO_2_	[31]

In addition to the bands arising from soybean components, three bands arising from the environment were detected. The tiny band between 4000 and 3500 cm^–1^ is attributable to water-vapor O-H stretching, and the other two bands correspond to carbon dioxide: O-C-O stretching at 2442–2208 cm^–1^ and O-C-O bending at 914–400 cm^–1^ [31]. As listed in Table 1 and shown in Fig 1, the peaks associated with lipids (2925, 2854, 1745, and 1456 cm^–1^) and proteins (3304, 1645, 1538, and 1239 cm^–1^) could be clearly discriminated.

### Determination of normalization and scaling methods

To determine the optimal normalization and scaling methods, permutation tests were carried out using two components. The normalization methods used were area normalization, amide normalization, min-max normalization, and vector normalization. Two types of scaling methods were employed: unit variance (UV) and Pareto scaling.

The permutation parameters of the PLS-DA models are listed in Table 2. The R^2^Y and Q^2^Y values, which indicate the model fit and predictability, respectively, range between 0 and 1.0. A PLS-DA model with a high R^2^Y value is regarded as providing a good fit to the data. A Q^2^Y value from 0.5 to 0.9 indicates good predictability, while one greater than 0.9 is considered to indicate excellent predictability. Permutation tests were performed to obtain the R^2^Y and Q^2^Y intercepts. The models were regarded as valid when the R^2^Y and Q^2^Y intercepts were less than 0.4 and 0.05, respectively [32].

**Table 2:** Selection of PLS-DA models according to various normalization and scaling methods for the differentiation of soybean samples.

Normalization methods	Scaling	R^2^Y	Q^2^Y		R^2^Y intercept	Q^2^Y intercept
**China vs. Korea**						
Area	UV	0.362	0.343		0.017	-0.094
	Par	0.200	0.194		0.025	-0.080
Amide	UV	0.534	0.317		-0.001	-0.078
	Par	0.082	0.071		0.017	-0.063
Min-max	UV	0.398	0.347		0.018	-0.072
	Par	0.104	0.102		0.031	-0.070
Vector (first)	UV	0.812	0.802		0.157	-0.129
	Par	0.772	0.762		0.079	-0.113
Vector (second)	UV	0.938	0.912		0.337	-0.187
	Par	0.883	0.861		0.251	-0.163
**Three groups of Chinese provinces**			
Area	UV	0.390	0.384		-0.015	-0.128
	Par	0.408	0.405		-0.048	-0.155
Amide	UV	0.373	0.364		-0.031	-0.147
	Par	0.383	0.378		-0.022	-0.119
Min-max	UV	0.349	0.339		-0.026	-0.126
	Par	0.328	0.320		0.002	-0.101
Vector (first)	UV	0.495	0.467		0.050	-0.176
	Par	0.456	0.444		0.024	-0.150
Vector (second)	UV	0.747	0.701		0.133	-0.220
	Par	0.606	0.563		0.089	-0.181
**Three groups of Korean provinces**			
Area	UV	0.330	0.278		0.042	-0.185
	Par	0.336	0.326		0.000	-0.115
Amide	UV	0.312	0.281		0.039	-0.109
	Par	0.302	0.249		-0.002	-0.138
Min-max	UV	0.352	0.334		0.011	-0.158
	Par	0.353	0.309		-0.005	-0.144
Vector (first)	UV	0.588	0.545		0.101	-0.156
	Par	0.527	0.494		0.083	-0.182
Vector (second)	UV	0.809	0.771		0.220	-0.255
	Par	0.809	0.771		0.220	-0.255

Two components were used to analyze in all PLS-DA models. The Chinese provinces are the northeastern, eastern, and southeastern regions. Korean provinces are the upper, left-side, and right-side regions. PLS-DA: partial least square discrimination analysis, UV: unit variance, and Par: Pareto.

The optimal PLS-DA models were selected after comparing the R^2^Y and Q^2^Y values. The optimal normalization and scaling methods for the model involved applying vector normalization after the second differentiation and UV scaling methods. The results are presented in Table 2, which indicates that this procedure yielded highest R^2^Y and Q^2^Y values of 0.938 and 0.912, respectively, for the comparison between Chinese and Korean soybean samples, of 0.747 and 0.701 for the comparison of Chinese soybean samples, and of 0.809 and 0.771 for the comparison of Korean soybean samples.

Table 2 indicates that both the R^2^Y and Q^2^Y values were highest when using the vector normalization method, which is possibly due to the derivative process used in vector normalization revealing minute differences between similar spectra [33]. This hypothesis is supported by the use of second derivatives allowing better discrimination of the minute differences in the FT-IR spectra compared to using first derivatives.

### Development of a PLSR model for determining the origin of soybeans using appropriate wave-number selection

PLSR can be employed to construct a prediction model for the origin of soybeans. Soybean-origin PLSR models were developed by applying suitable vector normalization after a second differentiation and UV scaling, and using two components. Apart from normalization, scaling methods, and the number of components, the VIP cutoff value was used to establish the most-precise prediction model. Training sets (six replicates) and a test set (one replicate) were prepared to construct the PLSR models. Both sets were used to obtain root-mean-square error (RMSE) values, including the root-mean-square error of estimation (RMSEE) and the root-mean-square error of prediction (RMSEP). RMSEE can be obtained from training sets, and its value is used to evaluate the accuracy of a PLSR model. RMSEP, which can be obtained from the test set, is employed to assess the predictability of PLSR models. These RMSE values range from 0 to 1, with smaller values indicating higher model accuracy and predictability.

Because FT-IR spectra may be affected by environmental factors such as water vapor and carbon dioxide, PLSR models constructed using different wave-number regions were compared to identify the best prediction model. Three wave-number regions were used to obtain prediction models: 4000–400 cm^–1^, 4000–400 cm^–1^ excluding the water vapor and carbon dioxide regions, and 2000–400 cm^–1^.

As listed in Tables 3–5, numerous VIP cutoff values were used to select better prediction models based on the RMSEP values. The permutation parameters of the PLSR models for comparing Chinese and Korean soybeans are listed in Table 3, while those for comparisons of Chinese soybeans and of Korean soybeans are listed in Tables 4 and 5, respectively.

**Table 3:** List of permutation parameters of the PLSR models obtained using variables selected by vector normalization applied after the second differentiation, UV scaling, and with various VIP cutoff values using different wavenumber areas for the comparison of Chinese and Korean soybeans.

Normalization method	VIP cutoff	Total wavenumber	RMSEE	RMSEP	R^2^Y	Q^2^Y	R^2^Y intercept	Q^2^Y intercept
**4000–400 cm**^**-1**^								
**Vector (second)**	total	7469	0.123	0.146	0.938	0.912	0.342	-0.212
	1.0	2297	0.148	0.176	0.909	0.898	0.193	-0.217
	1.2	1636	0.156	0.193	0.900	0.889	0.156	-0.169
	1.5	951	0.164	0.216	0.889	0.877	0.116	-0.125
	1.8	542	0.175	0.229	0.873	0.860	0.059	-0.145
	1.9	443	0.177	0.232	0.870	0.857	0.048	-0.124
	2.0	359	0.172	0.228	0.877	0.865	0.052	-0.121
**4000–400 cm**^**-1**^ **except water vapor, carbon dioxide region**								
**Vector (second)**	Total	7469	0.108	0.120	0.952	0.935	0.297	-0.163
	1.0	1868	0.127	0.139	0.933	0.927	0.197	-0.166
	1.2	1313	0.135	0.147	0.924	0.919	0.152	-0.129
	1.5	729	0.142	0.155	0.917	0.911	0.148	-0.147
	1.8	403	0.190	0.217	0.891	0.886	0.064	-0.132
	1.9	306	0.167	0.188	0.885	0.881	0.048	-0.132
	2.0	252	0.167	0.193	0.884	0.880	0.047	-0.123
**2000–400 cm**^**-1**^								
**Vector (second)**	total	7469	0.151	0.146	0.906	0.886	0.272	-0.189
	1.0	1049	0.166	0.164	0.886	0.879	0.105	-0.181
	1.2	780	0.175	0.174	0.873	0.866	0.065	-0.141
	1.5	441	0.187	0.185	0.855	0.849	0.009	-0.158
	1.8	228	0.197	0.202	0.840	0.834	0.009	-0.146
	1.9	181	0.196	0.203	0.841	0.837	0.008	-0.109
	2.0	150	0.200	0.208	0.834	0.830	0.017	-0.123

Two components were used to analyze in all PLSR models. VIP: variable influence on projection. UV: unit variance.

**Table 4:** List of permutation parameters of the PLSR models obtained using variables selected by vector normalization applied after the second differentiation, UV scaling, and with various VIP cutoff values using different wavenumber areas for the comparison of the three groups of Chinese provinces.

Normalization method	VIP cutoff	Total wavenumber	RMSEE	RMSEP	R^2^Y	Q^2^Y	R^2^Y intercept	Q^2^Y intercept
**4000–400 cm**^**-1**^								
**Vector (second)**	total	7469	0.270	0.389	0.898	0.867	0.369	-0.217
	1.0	3176	0.333	0.396	0.844	0.824	0.181	-0.173
	1.2	2199	0.368	0.411	0.810	0.794	0.182	-0.159
	1.5	517	0.323	0.368	0.854	0.840	0.233	-0.184
	1.8	64	0.382	0.528	0.795	0.758	0.189	-0.178
	1.9	36	0.497	0.720	0.654	0.594	0.177	-0.140
	2.0	17	0.580	0.759	0.529	0.479	0.063	-0.195
**4000–400 cm**^**-1**^ **except water vapor, carbon dioxide region**								
**Vector (second)**	Total	7469	0.255	0.317	0.909	0.884	0.360	-0.141
	1.0	2718	0.344	0.381	0.834	0.815	0.212	-0.152
	1.2	1783	0.380	0.402	0.798	0.782	0.151	-0.156
	1.5	290	0.262	0.293	0.904	0.891	0.258	-0.143
	1.8	51	0.418	0.594	0.755	0.715	0.170	-0.181
	1.9	28	0.501	0.719	0.648	0.601	0.078	-0.228
	2.0	14	0.595	0.729	0.504	0.466	0.062	-0.190
**2000–400 cm**^**-1**^								
**Vector (second)**	total	7469	0.310	0.384	0.865	0.838	0.298	-0.185
	1.0	1567	0.359	0.413	0.820	0.801	0.134	-0.193
	1.2	969	0.386	0.423	0.791	0.777	0.090	-0.166
	1.5	110	0.276	0.339	0.893	0.884	0.166	-0.266
	1.8	17	0.279	0.341	0.891	0.882	0.201	-0.215
	1.9	6	0.273	0.356	0.895	0.886	0.201	-0.227
	2.0	1	0.273	0.366	0.896	0.886	0.202	-0.230

Two components were used to analyze in all PLSR models. The Chinese provinces are grouped into northeastern, eastern, and southeastern regions. VIP: variable influence on projection. UV: unit variance.

**Table 5:** List of permutation parameters of the PLSR models obtained using variables selected by vector normalization applied after the second differentiation, UV scaling, and with various VIP cutoff values using different wavenumber areas for the comparison of the three groups of Korean provinces.

Normalization method	VIP cutoff	Total wavenumber	RMSEE	RMSEP	R^2^Y	Q^2^Y	R^2^Y intercept	Q^2^Y intercept
**4000–400 cm**^**-1**^								
**Vector (second)**	total	7469	0.138	0.199	0.971	0.947	0.527	-0.318
	1.0	3054	0.197	0.249	0.940	0.922	0.327	-0.335
	1.2	1934	0.234	0.291	0.915	0.897	0.352	-0.263
	1.5	616	0.237	0.277	0.914	0.895	0.346	-0.234
	1.8	118	0.294	0.289	0.867	0.848	0.203	-0.274
	1.9	70	0.342	0.386	0.820	0.783	0.202	-0.236
	2.0	40	0.368	0.467	0.792	0.729	0.150	-0.225
**4000–400 cm**^**-1**^ **except water vapor, carbon dioxide region**								
**Vector (second)**	Total	7469	0.158	0.215	0.961	0.940	0.512	-0.267
	1.0	2363	0.134	0.192	0.972	0.965	0.368	-0.232
	1.2	1470	0.138	0.189	0.971	0.965	0.332	-0.199
	1.5	482	0.129	0.170	0.974	0.968	0.350	-0.197
	1.8	119	0.113	0.192	0.980	0.967	0.297	-0.172
	1.9	73	0.137	0.257	0.971	0.943	0.269	-0.196
	2.0	45	0.148	0.293	0.966	0.937	0.215	-0.242
**2000–400 cm**^**-1**^								
**Vector (second)**	total	7469	0.186	0.292	0.947	0.922	0.350	-0.330
	1.0	1414	0.176	0.284	0.952	0.944	0.291	-0.201
	1.2	794	0.179	0.278	0.951	0.944	0.230	-0.229
	1.5	200	0.120	0.237	0.978	0.965	0.298	-0.287
	1.8	53	0.190	0.358	0.944	0.931	0.176	-0.299
	1.9	28	0.285	0.373	0.875	0.838	0.147	-0.213
	2.0	19	0.317	0.319	0.845	0.809	0.092	-0.265

Two components were used to analyze in all PLSR models. The Korean provinces are grouped into upper, left-side, right-side regions. VIP: variable influence on projection. UV: unit variance.

The PLSR models were compared to identify the PLSR models that satisfied the R^2^Y and Q^2^Y intercepts and had the lowest RMSEP values. The FT-IR spectral region between 4000 and 400 cm^–1^ that excluded the water vapor and carbon dioxide regions was the best. The PLSR model that did not apply a VIP cutoff value was selected for the prediction model presented in Table 3 for discriminating Chinese and Korean soybeans because it had the smallest RMSEP value (= 0.120). Table 4 indicates that the PLSR model with a VIP cutoff value of 1.5 was the optimal prediction model for discriminating Chinese soybeans, having an RMSEP value of 0.293, while Table 5 indicates that the PLSR model for discriminating Korean soybeans had the lowest RMSEP value of 0.170 for a VIP cutoff value of 1.5.

**Fig 5:**
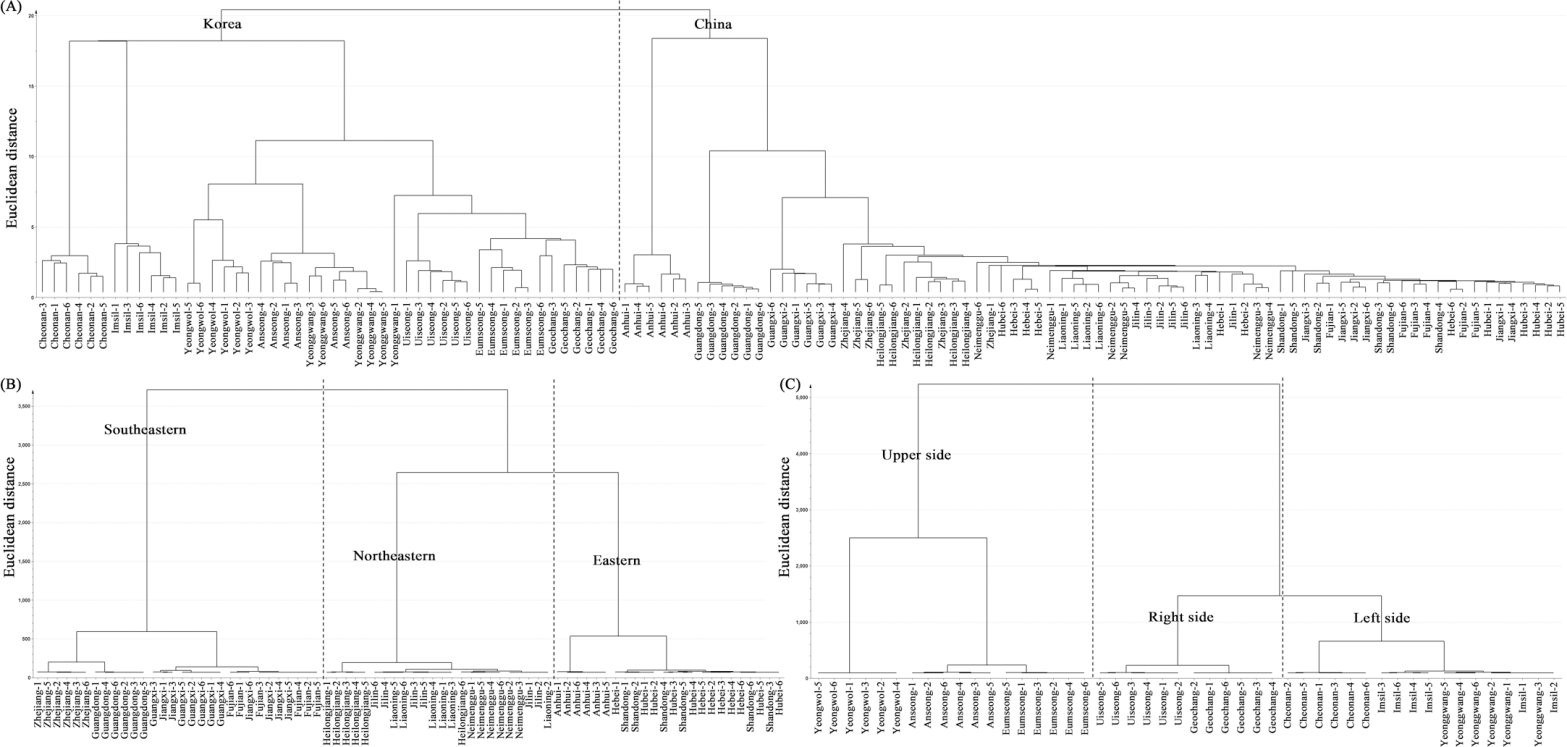
Hierarchical cluster analysis derived from the most suitable prediction models for the discrimination of soybean samples. (A) Chinese vs. Korean soybean samples (single linkage), (B) discrimination of Chinese soybean samples (Ward), and (C) discrimination of Korean soybean samples (Ward).

HCA dendrograms were constructed to evaluate the similarity of the samples using the optimal PLSR models for discriminating the soybean samples. As shown in Fig 5A, the Chinese and Korean soybean samples could be clearly discriminated using the single linkage method. Fig 5B shows that the soybean samples from the northeastern and eastern provinces of Chinese were clustered in the same clade using Ward’s method, whereas those from the southeastern provinces comprised the other clade. As shown in Fig 5C, three regions (upper, right side, and left side) were clustered using Ward’s method. Because the soybeans from the right- and left-side provinces appeared to be similar, the Korean provinces could be simply divided into upper and lower provinces. This result suggests that Chinese and Korean soybean samples can be discriminated by latitude-dependent climatic factors without consideration of the plant variety.

### Practical application of the PLSR model for predicting the origin of soybeans

The results presented in Tables 3–5 indicate that it is not only possible to discriminate between Chinese and Korean soybeans but also to identify the region in which soybeans have been cultivated. There is a wide diversity of soybean varieties used in China and Korea, but the present results indicate that it is possible to determine the origin of soybeans without considering their variety.

Our results indicate that it is possible to discriminate where soybeans originate from because they reflect regional characteristics. The soybean samples from China could be divided into those from the northeastern provinces (Neimenggu, Heilongjiang, Jilin, and Liaoning), Huang-Huai-Hai (Hebei, Shandong, and Anhui), Yangtze River (Hubei), and the southeastern provinces (Zhejiang, Jiangxi, Fujian, Guangdong, and Guangxi). If Huang-Huai-Hai and the Yangtze River region are considered to be the same province (due to their geographical proximity), the separations of the Chinese provinces are highly consistent with the predictions based on dividing the soybean regions into the northeastern, eastern, and southeastern provinces. The samples from South Korea were divided into those from the central provinces (Gyeonggi-do, Gangwon-do, Chungcheongbuk-do, and Chungcheongnam-do), Honam provinces (Jeollabuk-do and Jeollanam-do), and Youngnam provinces (Gyeongsangbuk-do and Gyeongsangnam-do). The results in Table 5 indicate that it was possible to separate three provinces (upper, left side, and right side) if Chungcheongnam-do (a central province) was grouped with Honam province.

**Fig 6:**
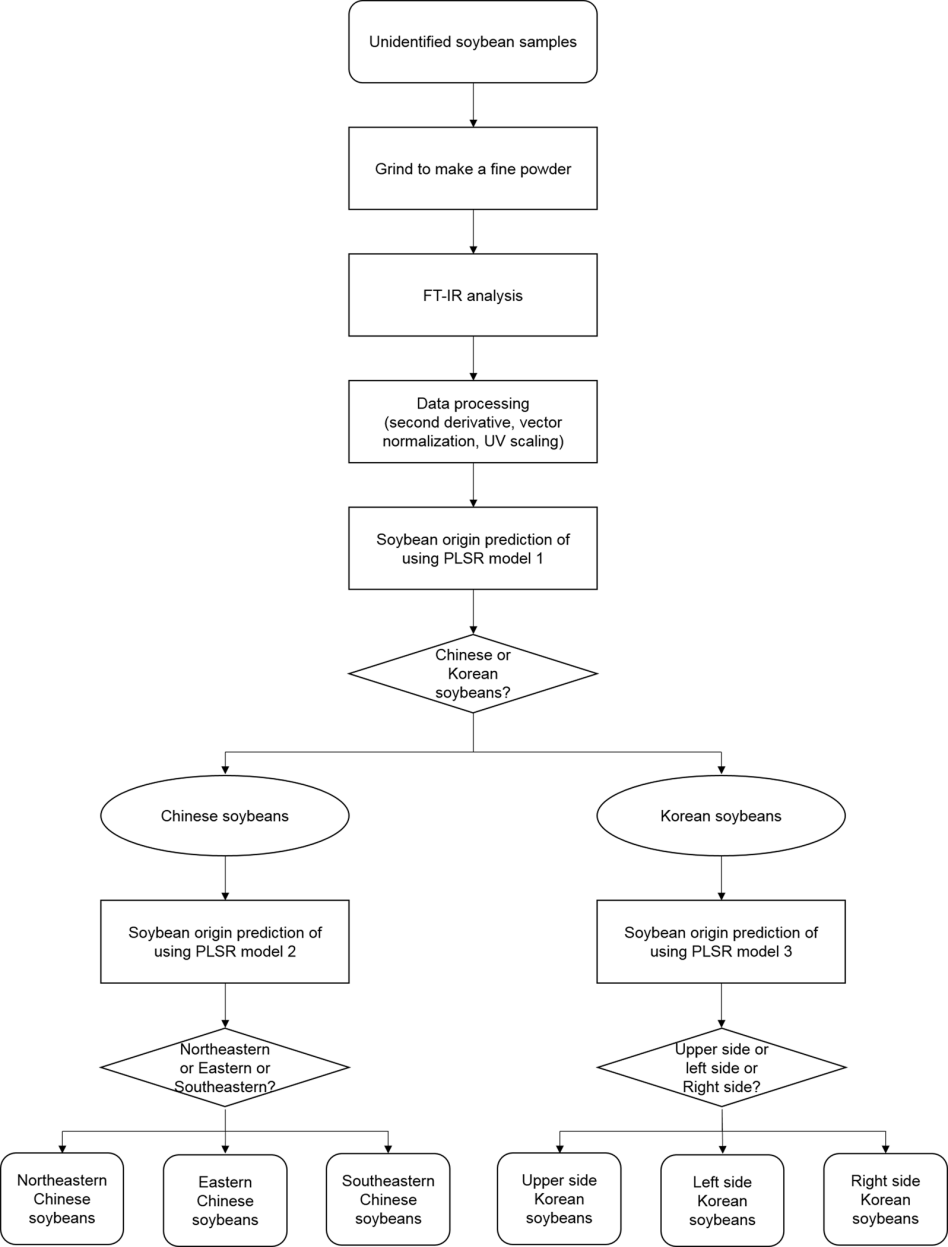
Flow chart for discrimination of unidentified soybean origin using FT-IR.

The flow chart in Fig 6 shows a method for discriminating the country of origin using prediction models when identifying unknown soybean samples. It is unclear where some of the soybeans available in Korean markets originate from, often because they are substituted by cheaper Chinese soybeans. The present results indicate that the flow chart in Fig 6 can be used to verify the origin of any suspect soybeans. Moreover, in addition to discriminating between Chinese and Korean soybeans, it is possible to discriminate between various production regions in a single country. Our flow chart can be applied to identify the original location of cultivation and detect the adulteration of the cultivation origin of soybeans.

## Conclusion

In this study we investigated whether FT-IR spectroscopy analysis can be combined with multivariate statistical analysis to predict the country of origin of soybean samples. This is the first study to discriminate the origin of soybeans using various factors including scaling methods, normalization methods, VIP cutoff, and wave-number region. These particular factors were selected since they allow the origin of soybeans to be determined easily and precisely. Our experimental results showed that this method could discriminate not only the country of origin but also the region of production within a country. The best PLSR prediction models for discriminating the origins employed UV scaling, vector normalization (second derivative), and the wave-number region from 4000 to 400 cm^–1^ excluding the water vapor and carbon dioxide regions. The PLSR prediction model for discriminating the country of origin (Chinese vs. Korean soybeans) was more precise when a VIP cutoff was not used. When the PLSR prediction models were constructed using a VIP cutoff within a single country, a VIP cutoff value of 1.5 was found to be optimal for discriminating the origin of soybeans.

Various soybean varieties and landraces are provided and grown worldwide according to the demands of both growers and consumers. Soybean cultivars reportedly have a short market life; for example, 54% of the cultivars submitted to the Varietal Information Program for Soybeans (the program supported by the Illinois Soybean Association of the US) are new [34]. In addition, various types of soybean seed are utilized in the production of products such as meal, tofu, soymilk, and edamame, and these seeds can exhibit various differences such as in their texture, color, and hilum characteristics. It is also thought that soybean germ plasm has been exchanged internationally. Therefore, PLSR models for predicting or differentiating soybean samples should be updated regularly (at least every 4–5 years) by sampling and analyzing the available samples using FT-IR spectroscopy. We suggest the application of additional objective criteria for the differentiation of various soybean seeds (varieties and landraces), such as the basic and novel protocols for differentiation and prediction as used in this study based on the optimization of preprocessing methods using FT-IR spectroscopy.

The practical application of these methods will require further studies using soybean samples from other countries. Once soybeans from many countries have been investigated, it might be possible to discriminate the countries of origin of unidentified soybean samples by using FT-IR spectroscopy combined with multivariate statistical analysis.

## Supporting information

S1 Table The provinces, cities, and geographic coordinates of soybean samples harvested in 2016 from Republic of Korea and China. (DOCX)
