# Supplementary material for: Discrimination and prediction of the origin of Chinese and Korean soybeans using Fourier transform infrared spectrometry (FT-IR) with multivariate statistical analysis
Source: PLoS One. 2018 Apr 24;13(4):e0196315. doi: 10.1371/journal.pone.0196315 (PMC5916519; doi:10.1371/journal.pone.0196315)
Supplement: S1 Table — (DOCX) [file pone.0196315.s001.docx]

**Supporting information**

**S1 Table. The provinces, cities, and geographic coordinates of soybean samples harvested in 2016 from Republic of Korea and China.**

|  | **No.** | **Province** | **City** | **Geographic coordinate** |
| --- | --- | --- | --- | --- |
| Korean samples | 1 | Gyeonggi-do | Anseong | N37°, E127° |
|  | 2 | Gangwon-do | Yeongwol | N37°, E128° |
|  | 3 | Chungcheongbuk-do | Eumseong | N36°, E127° |
|  | 4 | Chungcheongnam-do | Cheonan | N36°, E127° |
|  | 5 | Jeollabuk-do | Imsil | N35°, E127° |
|  | 6 | Jeollanam-do | Yeonggwang | N35°, E126° |
|  | 7 | Gyeongsangbuk-do | Uiseong | N36°, E128° |
|  | 8 | Gyeongsangnam-do | Geochang | N35°, E127° |
| Chinese samples | a | Neimenggu | Ulanhot | N40°, E111° |
|  | b | Heilongjiang | Harbin | N45°, E126° |
|  | c | Jilin | Meihekou | N42°, E125° |
|  | d | Liaoning | Dandong | N40°, E124° |
|  | e | Hebei | Shijiazhuang | N38°, E114° |
|  | f | Shandong | Jining | N35°, E116° |
|  | g | Anhui | Huaibei | N33°, E116° |
|  | h | Hubei | Huangshi | N30°, E115° |
|  | i | Zhejiang | Jiaxing | N30°, E120° |
|  | j | Jiangxi | Jiujiang | N29°, E115° |
|  | k | Fujian | Longyan | N25°, E117° |
|  | l | Guangdong | Shaoguan | N24°, E113° |
|  | m | Guangxi | Hechi | N24°, E108° |

*Korean soybean samples were provided by the National Agricultural Products Quality Management Service.
